# Supplementary material for: Predictors of one-year mortality following hip fracture surgery in elderly
Source: PeerJ. 2023 Sep 8;11:e16008. doi: 10.7717/peerj.16008 (PMC10494834; doi:10.7717/peerj.16008)
Supplement: Supplemental Information 1 [file peerj-11-16008-s001.docx]

**Completed STROBE checklist for cross-sectional studies and analysis plan information**

This checklist was elaborated using formal items recommended for cross-sectional studies from STROBE statement (https://www.strobe-statement.org).

|  | Item No | Recommendation | Respected ? | Comments and quotes | |
| --- | --- | --- | --- | --- | --- |
| **Title and abstract** | 1 | (*a*) Indicate the study’s design with a commonly used term in the title or the abstract | Yes | | Study design is indicated in the Methods/Findings section of the abstract  “A retrospective study was conducted on 275 elderly patients who underwent hip fracture surgery in a tertiary hospital between January 2018 and January 2022” |
|  |  | (*b*) Provide in the abstract an informative and balanced summary of what was done and what was found | Yes | | These information are stated in the study abstract (study objective described, method and results described) |
| Introduction | | |  | |  |
| Background/rationale | 2 | Explain the scientific background and rationale for the investigation being reported | Yes | | Rationale and existing literature are stated in the introduction section |
| Objectives | 3 | State specific objectives, including any prespecified hypotheses | Yes | | A statement at the end of the introduction specifies the specific goals and objectives.  “The aims of this study were two-fold:  1) This study aimed to investigate the effects of admission and postoperative levels of RDW, albumin, and RDW/Albumin (RA) ratio on predicting 1-year mortality following hip fracture surgery. |
| Methods | | |  | |  |
| Study design | 4 | Present key elements of study design early in the paper | Yes | | Study design is stated in the first subsection of Methods. Key elements are all described in the methods.  “A retrospective single-centre study conducted….” |
| Setting | 5 | Describe the setting, locations, and relevant dates, including periods of recruitment, exposure, follow-up, and data collection | Mostly | | Setting, contexts, dates of inclusion, are fully described in the method section under “Setting” and “Study population” headlines page 4 and 5. ‘’Study design and patients’’ and ‘’Clinical data collection and outcome assessments’’ |
| Participants | 6 | (*a*) Give the eligibility criteria, and the sources and methods of selection of participants | Yes | | Study population is described is the method section (Study population headline), as well as selection criteria  “A retrospective single-centre study conducted between January 2018 and January 2022.We included patients 65 years of age or older (elderly) hospitalized in orthopedics and traumatology department from emergency department (ED) who were diagnosed with hip fractures. The study excluded elderly patients with hip fractures resulting from high energy trauma, such as motor vehicle accidents or firearm related injury, and pathological fractures, such as those caused by tumours.’’ |
| Variables | 7 | Clearly define all outcomes, exposures, predictors, potential confounders, and effect modifiers. Give diagnostic criteria, if applicable | Yes | | Box 1-Data on sociodemographics, including age, gender, ASA score and past medical histories, including hypertension, diabetes, cardiac disease, chronic obstructive pulmonary disease (COPD), chronic kidney disease, and malignancy were collected. Laboratory variables such as hemoglobin (HGB), hematocrit (HCT), RDW, and albumin were measured with the usual methods. All patients' laboratory data were collected on admission to ED and on their first postoperative day. The RA ratio was calculated for all patients as well. Radiographs performed in ED were analyzed to confirm the fracture type and identify the anatomic location in patients with hip trauma.  Box-2 Among the variables analyzed were the need for blood transfusions, the length of hospital stay (LOS) (days), the time between hospitalization and surgery (in or out of office hours), the length of postoperative ICU follow-up (day), complications in the ICU (Intubation, cardiopulmonary resuscitation, need for inotropes and vasopressors, multiple organ failure, presence of sepsis).  Box -3 Deaths within the first year following fracture hospitalization were defined as 1-year mortality. The national database system was used to confirm whether the patients were alive or dead. Death causes were not collected. A survivor group and a deceased group were formed from all of the study patients. Patients who survived after hip fractures for at least one year were defined as survivor group. The deceased group was defined as patients who died within one year after a hip fracture. |
| Data sources/ measurement | 8* | For each variable of interest, give sources of data and details of methods of assessment (measurement). Describe comparability of assessment methods if there is more than one group | Yes | | Data collection and measurement was the same for all variables, and is described in the methods section. Demographic, clinical laboratory and radiologic data were obtained from the hospital information management system and mortality data were obtained from the national database.” |
| Bias | 9 | Describe any efforts to address potential sources of bias | Yes | | We notably tried to reduce bias by excluding suspect cases. ‘’The study excluded elderly patients with hip fractures resulting from high energy trauma, such as motor vehicle accidents or firearm related injury, and pathological fractures, such as those caused by tumours. |
| Study size | 10 | Explain how the study size was arrived at | Yes | | The method describes the inclusion of study between 2018 and 2022. Since the study was retrospective, patients who met the inclusion criteria on the specified date were included. |
| Quantitative variables | 11 | Explain how quantitative variables were handled in the analyses. If applicable, describe which groupings were chosen and why | Yes | | Definitions of all categories for variables are presented in Box 1  “Box 1: “A survivor group and a deceased group were formed from all of the study patients. Patients who survived after hip fractures for at least one year were defined as survivor group. The deceased group was defined as patients who died within one year after a hip fracture. |
| Statistical methods | 12 | (*a*) Describe all statistical methods, including those used to control for confounding | Yes | | These are described in the method section.  “The data from the hospital information management system  and the national database, already available in Excel software, were pooled and statistical analysis was performed using 28.0 for Mac (SPSS, Chicago, IL, USA). Missing values were not inferred. Analysis of the data was conducted using SPSS 28.0 for Mac (SPSS, Chicago, IL, USA). A Shapiro-Wilk test and histogram were used to determine the normal distribution of variables. A descriptive statistic consists of a mean ± standard deviation or median (interquartile range IQR) for continuous variables, and percentages and counts for nominal and categorical variables. The Fisher's exact test, Chi-Square (χ2) test, Student's t-test, and the Mann-Whitney U test were used for univariate analysis. Using odds ratios (OR) and 95% confidence intervals (CI), binary logistic regression was used to verify the results of the univariate analysis. Statistical significance was determined by an alpha value of 0.05 (in the multivariate analysis). |
|  |  | (*b*) Describe any methods used to examine subgroups and interactions | Yes | | This is described in the method section |
|  |  | (*c*) Explain how missing data were addressed | Yes | | This is described in the method section.  “Missing values were not inferred” |
|  |  | (*d*) If applicable, describe analytical methods taking account of sampling strategy | N/A | | Non applicable |
|  |  | (*e*) Describe any sensitivity analyses | N/A | | Non applicable |
| Results | | |  | |  |
| Participants | 13* | (a) Report numbers of individuals at each stage of study—eg numbers potentially eligible, examined for eligibility, confirmed eligible, included in the study, completing follow-up, and analysed | Yes | | This is described at the beginning of result section  ‘’A total of 275 patients were included in the study. It was found that the mortality rate at 1 year was 34.7%. As shown on Table 1…’’ |
|  |  | (b) Give reasons for non-participation at each stage | Yes | | This is described in the method section  ‘The study excluded elderly patients with hip fractures resulting from high energy trauma, such as motor vehicle accidents or firearm related injury, and pathological fractures, such as those caused by tumours.’’ Because these conditions would affect the mortality rate, they were not included in the study, and data on age, gender, etc. were not analyzed. |
|  |  | (c) Consider use of a flow diagram | N/A | | Use of a flow diagram was not deemed appropriate |
| Descriptive data | 14* | (a) Give characteristics of study participants (eg demographic, clinical, social) and information on exposures and potential confounders | Yes | | Table 1 describes the participants and groups included.  “Table 1: Comparison of patient demographic data and co-morbid diseases by groups” |
|  |  | (b) Indicate number of participants with missing data for each variable of interest | Yes | | The total numbers of recorded data for each variable are stated in variable headline of each table |
| Outcome data | 15* | Report numbers of outcome events or summary measures | Yes | | All numbers are reported in Tables |
| Main results | 16 | (*a*) Give unadjusted estimates and, if applicable, confounder-adjusted estimates and their precision (eg, 95% confidence interval). Make clear which confounders were adjusted for and why they were included | Mostly | | All adjusted estimates, and 95% confidence intervals, are reported in Table 4. Non-adjusted estimates were not displayed in the interest of table clarity. |
|  |  | (*b*) Report category boundaries when continuous variables were categorized | Yes | | Category boundaries are displayed in variable headings in the tables, where applicable |
|  |  | (*c*) If relevant, consider translating estimates of relative risk into absolute risk for a meaningful time period | N/A | | N/A |
| Other analyses | 17 | Report other analyses done—eg analyses of subgroups and interactions, and sensitivity analyses | N/A | | N/A |
| Discussion | | |  | |  |
| Key results | 18 | Summarise key results with reference to study objectives | Yes | | Key results are described at the beginning of discussion section (page 6), and then through other paragraphs showing its main operational implications. They also are summarized in the conclusion |
| Limitations | 19 | Discuss limitations of the study, taking into account sources of potential bias or imprecision. Discuss both direction and magnitude of any potential bias | Yes | | Description of limitations is done under “Limitations” heading in the discussion. |
| Interpretation | 20 | Give a cautious overall interpretation of results considering objectives, limitations, multiplicity of analyses, results from similar studies, and other relevant evidence | Yes | | References were added where possible, and discussed. Limitations were taken into account in the discussion. |
| Generalisability | 21 | Discuss the generalisability (external validity) of the study results | Yes | | Study results were deemed for clinical planning and decision-making in the elderly with hip fractures and discussed as such. |
| Other information | | |  | |  |
| Funding | 22 | Give the source of funding and the role of the funders for the present study and, if applicable, for the original study on which the present article is based | N/A | | This study was not funded by any grant. |
| **Additional information on analysis plan** | | |  | |  |
| “For observational studies, authors are required to clearly specify (a) What specific hypotheses the researchers intended to test, and the analytical methods by which they planned to test them; (b) What analyses they actually performed; and (c) When reported analyses differ from those that were planned, authors must provide transparent explanations for differences that affect the reliability of the study's results.” PLoS Med submission guidelines | | | (a) The hypotheses and goal of this study is stated page 4 L136-138. The analysis was first planned to include odds ratio calculation for the survivors-deceased adjusted comparison by context type for variables affecting 1-year mortality  (b) Odds ratio analysis was fully performed as planned.  (c) The reliability of the study results was not affected | | |
| “If a prospective analysis plan (from the study's funding proposal, IRB or other ethics committee submission, study protocol, or other planning document written before analyzing the data) was used in designing an observational study, authors must include the relevant prospectively written document with the manuscript submission for access by editors and reviewers and eventual publication alongside the accepted paper. If no prospectively written document exists, authors should explain how and when they determined the analyses being reported.” PLoS Med submission guidelines | | | This study had a retrospective design. But IRB and or other ethics committee submission written before analyzing the data)  The analyses were agreed upon with all authors of the study at the beginning of the study and then, after the database was established and the factors affecting mortality according to the living and deceased groups were examined. | | |

*Give information separately for exposed and unexposed groups.
